# Genomic analysis of bifunctional Class C-Class D β-lactamases in environmental bacteria

**DOI:** 10.1590/0074-02760180098

**Published:** 2018-05-28

**Authors:** Melise Chaves Silveira, Marcos Catanho, Antônio Basílio de Miranda

**Affiliations:** 1 Fundação Oswaldo Cruz-Fiocruz, Instituto Oswaldo Cruz, Laboratório de Biologia Computacional e Sistemas, Rio de Janeiro, RJ, Brasil; 2 Fundação Oswaldo Cruz-Fiocruz, Instituto Oswaldo Cruz, Laboratório de Genômica Funcional e Bioinformática, Rio de Janeiro, RJ, Brasil

**Keywords:** bifunctional β-lactamase, antibiotic resistance, health surveillance

## Abstract

β-lactamases, which are found in several bacterial species and environments, are the main cause of resistance to β-lactams in Gram-negative bacteria. In 2009, a protein (LRA-13) with two β-lactamase domains (one class C domain and one class D domain) was experimentally characterised, and an extended action spectrum against β-lactams consistent with two functional domains was found. Here, we present the results of searches in the non-redundant NCBI protein database that revealed the existence of a group of homologous bifunctional β-lactamases in the genomes of environmental bacteria. These findings suggest that bifunctional β-lactamases are widespread in nature; these findings also raise concern that bifunctional β-lactamases may be transferred to bacteria of clinical importance through lateral gene transfer mechanisms.

β-lactamases are part of a large group of diverse and widely distributed enzymes, encoded by genes located on both the chromosome and on mobile genetic elements (Bush 2001, Srivastava et al. 2014). Bacteria containing β-lactamases have been found in a wide range of environmental conditions, including soil, water and in human and animal microbiota (Allen et al. 2009, Gibson et al. 2015, Fróes et al. 2016). The production of bacterial β-lactamases is the main cause of β-lactam resistance in Gram-negative bacteria (Bush 2001), and it is essential to know their spectrum of action and distribution (Bush 2001, Gibson et al. 2015).

A few years ago, a novel β-lactamase, LRA-13, was identified in the metagenome of uncultured bacteria isolated from Alaskan soil (Allen et al. 2009). LRA-13 contains two serine-β-lactamase domains - one belonging to class C and one to class D. The fusion of these domains expands the hydrolytic capacity of the protein beyond what either could display alone, thereby causing resistance to amoxicillin, ampicillin, cephalexin (class C) and carbenicillin (class D), as demonstrated experimentally (Allen et al. 2009). The identification of bifunctional β-lactamases in other bacterial species may indicate that more attention should be given to genes encoding this class of enzyme, particularly since lateral gene transfer events are common among prokaryotes (Soucy et al. 2015), and these genes could theoretically transfer to human bacterial pathogens.

To determine whether bifunctional β-lactamases are present in other bacterial species, we searched the non-redundant NCBI protein database (July 2017) utilising the BLAST programme (Altschul et al. 1997) to identify potential homologs of the LRA-13 enzyme. We identified nine putative homologs encoded in the genomes of nine different bacterial species or isolates (Table). The sequence of these nine proteins is highly conserved between the nine species (≥ 94%) and align closely with the reference sequence of LRA-13 (≥ 65%). All nine proteins have two complete characteristic domains of class C (COG1680, PRK11289) and one of class D (COG2602) according to the Conserved Domain Database (CDD, Batch CD-search tool) (Marchler-Bauer et al. 2017). In addition, these proteins display characteristic active site patterns of both class C (PS00336) and class D (PS00337) domains according to PROSITE (Sigrist et al. 2012), including the serine (S) catalytic residue.

**TABLE:** Genomes, original annotation and genomic context of genes enconding bifunctional β-lactamases

		Bifunctional β-lactamase		Upstream gene product		Downstream gene product	
Strain	Accession	Annotation	Accession	Annotation	Accession	Annotation	Accession
Uncul. Bacterium BLR13	EU408352.1	LRA-13	ACH58991.1	resp. reg.	ACH58992.1	glycoside hydrolase	ACN58887.1
Janthinobacterium sp. HH01	NZ_AMWD01000002.1	class C BL	WP_008451281.1	class D BL	WP_008451277.1	transaldolase	WP_008451283.1
Massilia sp. Root418	LMEC01000020.1	hypot. protein	KQW93884.1	class D BL	KQW93885.1	diguanylate cyclase	KQW93883.1
Massilia sp. Root351	NZ_LMDJ01000033.1	class C BL	WP_082552146.1	class D BL	WP_057157847.1	diguanylate cyclase	WP_057157849.1
Massilia sp. CF038	FQWU01000002.1	class C BL	SHH20105.1	class D BL	SHH20059.1	hypothetical protein	SHH20125.1
Duganella sp. HH105	LRHV01000029.1	class C BL	OEZ55387.1	class D BL	OEZ55388.1	transaldolase	OEZ55386.1
Duganella sp. CF458	FOOF01000012.1	class D BL	SFG43659.1	class D BL	SFG43677.1	nitrite reductase	SFG43637.1
Duganella sp. Root198D2	NZ_LMIC01000034.1	class C BL	WP_082591432.1	class D BL	WP_082591444.1	nitrite reductase	WP_082507115.1
Duganella sp. Root336D2	NZ_LMDB01000002.1	class C BL	WP_082507116.1	class D BL	WP_082507139.1	nitrite reductase	WP_082507115.1
Duganella sp. Root1480D1	NZ_LMFZ01000003.1	class C BL	WP_082565248.1	class D BL	WP_082565235.1	nitrite reductase	WP_082565234.1

Uncul.: uncultured; BL: β-lactamase; hypot: hypothetical; resp. reg.: putative response regulator.

We then examined whether these bifunctional β-lactamases are encoded in genomic islands or near prophage sequences using IslandViewer 4 (Bertelli et al. 2017), which integrates four different genomic island prediction methods, and the PHAge Search Tool (PHAST) (Zhou et al. 2011), which identifies prophage sequences in bacterial genomes. Bifunctional β-lactamase was not encoded in genomic islands, and only the strain *Massilia* sp. Root351 showed the presence of an incomplete 8.4Kb prophage located approximately 3.4Kb downstream from the gene encoding a bifunctional β-lactamase.

The β-lactamases with fused domains found in this work were identified in the genomes of bacterial strains belonging to three distinct Gram-negative genera (Baldani et al. 2014): *Duganella* spp., *Janthinobacterium* sp. and *Massilia* sp. The original annotation of the gene products encoding these enzymes is either “class C β-lactamase” or “class D β-lactamase” (Table). In all cases, the upstream protein-coding gene is originally annotated as “class D β-lactamase”, and their products display a complete characteristic domain of class D (COG2602). Additionally, these products display complete or incomplete domains of methicillin resistance regulatory proteins BlaR1 and MecR1 (COG4219, cd07341), corresponding to the structure of the signal-transducing integral membrane protein that regulates the β-lactam resistance in the Gram-positive species *Staphylococcus aureus* (Wilke et al. 2004). Genes located downstream of the bifunctional β-lactamase vary among distinct bacterial genera and fall into four functional categories: “diguanylate cyclase”, “transaldolase”, “glycoside hydrolase” or “hypothetical” (Table). All *Massilia* strains harbor a gene encoding a transcriptional regulator which is upstream and inverted relative to the gene encoding the bifunctional β-lactamase. This transcriptional regulator mediates the expression of the regulatory protein BlaR1, which is also inverted in relation to the bifunctional β-lactamase (Figure).

**Figure:**
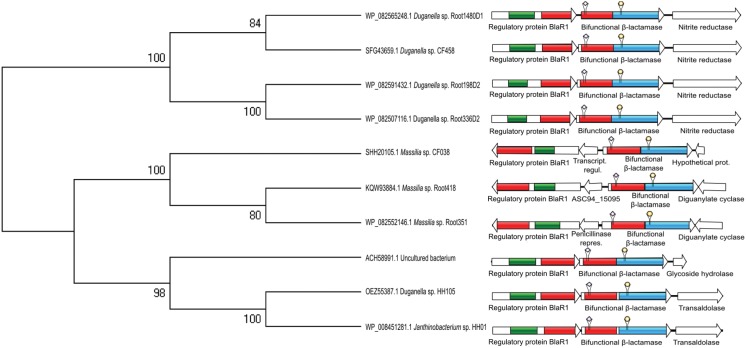
Phyletic pattern of genes encoding bifunctional β-lactamases, and their genomic organisation including surrounding genes. Left: a dendrogram representing the relationships between the bifunctional β-lactamases in this study. Right: a panel displaying the order and orientation of the genes encoding the bifunctional β-lactamases and surrounding genes. The boxes represent distinct domains: red, class D; green, MecR1/BlaR1; blue, class C. Arrows indicate gene orientation. Diamonds and circles above the bifunctional β-lactamases indicate the location of class D and class C active sites, respectively. Sequences were globally aligned using MAFFT version 7 (Katoh et al. 2017). The dendrogram was constructed with MEGA version 7 (Kumar et al. 2016), applying the NJ algorithm and 500 bootstrap replicates. The panel containing genes, domains and active sites was drawn using the IBS (Liu et al. 2015).

The strains *Janthinobacterium* sp. HH01 and *Duganella* sp. HH105 were isolated from an aquatic environment and exhibit an ampicillin resistance phenotype (Hornung et al. 2013, Haack et al. 2016). The strains *Duganella* sp. CF458 (Gp0136797) and *Massilia* sp. CF038 (Gp0136806) were isolated in 2016 from the root of a Populus tree in Tennessee, USA (NCBI BioProject PRJEB18228), while the other strains of *Duganella* sp. and *Massilia* sp. were isolated from the Arabidopsis root microbiota (Bai et al. 2015). These three genera belong to the family *Oxalobacteraceae* (*Betaproteobacteria* group); they are (supposedly) non-pathogenic to humans, animals and plants and are known for their antifungal effect (Yin et al. 2013, Haack et al. 2016). Bacteria from this family have few phenotypic differences, and their classification in distinct genera is mainly based on 16S rRNA gene sequencing (Kämpfer et al. 2007). Functional metallo-β-lactamases (class B) have already been described in *Janthinobacterium lividum* and *Massilia oculi* (Docquier et al. 2004, Gudeta et al. 2016). These genes are phylogenetically related and share common ancestors with acquired β-lactamases produced by clinical pathogens, which could have been acquired from members of *Oxalobacteraceae* (Gudeta et al. 2016).

According to Allen et al. (2009), the LRA-13 β-lactamase appears to be the result of an ancient natural fusion of genes encoding complete enzymes, not due to modern selective pressure caused by the extensive use of antibiotics. In two cases, the bifunctional β-lactamase sequences are virtually identical (*Duganella* sp. Root 198D2 vs. *Duganella* sp. Root 336D2, and *Duganella* sp. HH105 vs. *Janthinobacterium* sp. HH01) with 99% and 96% amino acid identity over their entire sequences, respectively. However, LRA-13 is not the only example of a bifunctional enzyme implicated in antibiotic resistance. Some aminoglycoside transferases are capable of conferring resistance to practically all antibiotics of this class via modifications to the antibiotic molecule at two different sites. However, unlike LRA-13, their origin appears to be recent and caused by the clinical (mis)use of aminoglycosides (Kim et al. 2007, Zhang et al. 2009).

The absence of genes encoding bifunctional β-lactamases in genomic islands or near prophage sequences, and the fact that these genes are shared among all currently sampled representatives of three genera belonging to the same family (*Oxalobacteraceae*), suggest that, similar to LRA-13, the gene fusion might have occurred naturally and long ago. Indeed, several benefits of bearing a bifunctional enzyme can be assumed, such as the concomitant mobilisation of two different functions, the potential for complementary and extended resistance, and the simultaneous selection of two enzymatic activities by the selective pressure exerted by a single antibiotic (Zhang et al. 2009).

The evidence presented here suggests that bifunctional β-lactamases are part of a new class of enzyme with potentially broad spectrums of action. The first reported enzyme within this class (LRA-13) was found in a non-cultivable bacterium from a remote soil sample, but proteins with the same characteristics can be found in different bacterial genera present in water, soil, and even sharing the same niche. To date, there is no evidence of a clinically significant role for bifunctional β-lactamases, but this possibility cannot be ignored. Chromosomal location and degree of sequence conservation suggest that these enzymes might be characteristic of the family *Oxalobacteraceae*. Since our knowledge of the environmental microbiota is far from complete, it is necessary to examine the eventual dissemination of these bifunctional β-lactamases to bacteria that are pathogenic to humans and other animals.
